# Stability analysis of sodium saccharin in fried nuts and seeds—Determination of sodium saccharin and *o*-sulfamoylbenzoic acid by HPLC

**DOI:** 10.3389/fnut.2025.1645604

**Published:** 2025-10-24

**Authors:** Li Mu, Qianqian Liu, Sijia Sun, Yuhang Liu, Zeyue Gao, Li Zhang, Ying Xu, Gang Li

**Affiliations:** ^1^College of Food Science and Engineering of ChangChun University, Changchun, Jilin, China; ^2^Jilin Province Product Quality Supervision and Inspection Institute, Changchun, Jilin, China

**Keywords:** sodium saccharin, *o*-sulfamoylbenzoic acid, high temperature frying, high performance liquid chromatography (HPLC), stability

## Abstract

Since the invention of synthetic sweeteners, there has been considerable debate regarding the safety of some of these sweeteners due to their poor thermal stability. Sodium saccharin is a synthetic sweetener used to modulate the flavor of fried nuts and seeds. The change in saccharin sodium during high-temperature frying was investigated, and a detection method for saccharin sodium and its frying product, *o*-sulfamoylbenzoic acid, was established using high-performance liquid chromatography (HPLC). Sodium saccharin will be completely decomposed at 190 °C for 40 min by frying. *O*-sulfamoylbenzoic acid is the decomposition product of sodium saccharin under high-temperature frying conditions. An HPLC method was developed for the simultaneous determination of sodium saccharin and *o*-sulfamoylbenzoic acid. When the UV wavelength was 264 nm, the mobile phase was an acetonitrile-0.1% formic acid aqueous solution with gradient elution, and the detection was performed using HPLC. The detection limit of sodium saccharin was 0.045 mg/kg, and the limit of quantification (LOQ) was 0.135 mg/kg. *O*-sulfamoylbenzoic acid showed a detection limit of 0.056 mg/kg and a limit of quantification of 0.167 mg/kg. The recovery rate of spiked sodium saccharin ranged from 105.67 to 112.16% with relative standard deviations (RSDs) between 0.24 and 1.39%. The recovery rate of *o*-sulfamoylbenzoic acid spiked between 98.67 and 108.16% with relative standard deviations (RSDs) ranging from 0.13 to 1.51%. We employed the established method to conduct application detection and analysis of a range of commercially available snack foods. Saccharin sodium was detected, while *o*-sulfamoylbenzoic acid was not found.

## 1 Introduction

Synthetic sweeteners are a class of food additives that are widely used in various industries and fields. However, there is skepticism about the safety of these sweeteners because their history of use is much shorter than that of natural sweeteners. Some synthetic sweeteners have potential cumulative hazards after consumption and may decompose under complex processing conditions in some foods (1). Sodium saccharin, a non-nutritive synthetic sweetener produced by the methylbenzene or phthalic anhydride process, is 300–500 times sweeter than sucrose (2). Stabilization of sodium saccharin decreases with increase in temperature. Sodium saccharin is not only used as a sweetener for food and beverages but also as a diagnostic drug for measuring blood circulation and as a pharmaceutical processing material (roxithromycin). It is also processed as a flavoring agent for toothpaste, cigarettes, and cosmetics. Additionally, it serves as a brightening flux for chrome plating (3). Industrial production of sodium saccharin began in the United States in 1910; it was introduced for consumption in the United States in the same year, and it gained popularity in the soft drink industry during the 1950s. On 27 October 2017, the World Health Organization's International Agency for Research on Cancer (IARC) published a list of carcinogens, with saccharin and its salts in the list of three groups of carcinogens. EFSA and the European Chemicals Agency (ECHA) consider sodium saccharin to be potentially hazardous by inhalation or in contact with the skin and eyes. GB2760-2024 (China) establishes the limit for the use of a sodium saccharin; compared to the preserved fruits and candied fruits class limit of 5 g/kg, foods that need to be heated and fried, such as shelled/cooked nuts and seeds, are limited to 1 g/kg. Additionally, for products that undergo repeated high-temperature heating and for seasonings, the limit is further decreased to 0.15 g/kg. In the United States, the FDA, along with France's regulatory agency, EFSA, and the European Chemicals Agency (ECHA) oversees non-high-temperature food heating processes (4, 5). Saccharin is not allowed to be used in food intended for infants and young children, people with weaker metabolic detoxification abilities, the elderly, pregnant women, as it may even lead to cancer or birth defects. China has implemented strict measures to ensure the safety of food processing (6). According to Frost and Sullivan, based on the latest 2023 international sodium saccharin data, nearly 60% is used in the daily chemical industry, and only 40% is used by food companies in their processing activities (7–10). Among these, approximately 70% is attributed to non-heated food processing, which includes products such as cold beverages, preserved fruits, and candied fruit production (National Bureau of Statistics of the People's Republic of China, Key Economic Indicators of Industrial Enterprises in the Food Manufacturing Industry, 2023).

*o*-sulfamoylbenzoic acid has many applications in the chemical industry. It is commonly used as an important intermediate in the production of dyes, drugs, and in organic synthesis. It is mainly used as a rubber antioxidant, vulcanization accelerator, and a chemical auxiliary in plastics and textiles. Additionally, it functions as a boiler water treatment agent, metal corrosion inhibitor, emulsifier, anticorrosive agent, antistatic agent, latex coagulant, petroleum additive, fungicide, pesticide, and dye intermediate. However, there is a potential risk of chronic and cumulative toxicity associated with its use, and currently, no relevant test methods are available to assess this risk (11, 12).

The available detection methods for sodium saccharin include liquid chromatography (GB 5009.28-2016), gas chromatography (GB 5009.28-2016), evaporative light scattering detection (13), ambient flame ionization mass spectrometry (14), silver nanorod array SERS (15), ion chromatography (12), and pulse polarography (11). Evaporative light-scattering detection has the advantage of detecting any sample that is less volatile than the mobile phase and can be connected to any liquid chromatograph for analysis (13). However, disadvantages are that the mobile phase must be volatile, and non-volatile buffer salts and surfactants cannot be used; the sensitivity is relatively low. Ambient flame ionization mass spectrometry can quantify sodium saccharin in a variety of food samples (14), but the detection cost is high, and there is a limitation on the detected substances. The silver nanorod array SERS method offers quick sample pre-treatment and high detection sensitivity, but its operation is complicated and the detection costs are high (15–17). Ion chromatography has strong specificity and high sensitivity and stability; however, when detecting target substances with large concentrations, samples need to be diluted. Ion chromatography has shortcomings such as low reproducibility and unstable instruments. Hannisdal (18) developed a rapid differential pulse polarography method for the determination of the synthetic sweeteners potassium acesulfame and saccharin in beverages. After methodological validation, it was found that the quantitative results were consistent with those of high-performance liquid chromatography (HPLC).

The “Tmall Food Industry Trend Analysis Report” jointly released by the First Financial Data Center and Tmall Food shows that the preference for small packages is growing among both female and male consumers (19). Young people and children like to eat snacks while playing and entertaining. Popular snacks include fried nuts, seeds, and peanuts. Many people prefer consuming fried foods with sodium saccharin, which has a sweet and tangy flavor. To reduce costs, some business owners reuse frying oil multiple times. Currently, there are few reports of high-temperature frying with sodium saccharin, and the safety of this additive is still under debate (20–23). In this study, the changes in the content of sodium saccharin were investigated during high-temperature frying, and it was identified that *o*-sulfamoylbenzoic acid was the primary decomposition product. Furthermore, a high-performance liquid chromatography (HPLC) method for the detection of sodium saccharin and *o*-sulfamoylbenzoic acid was established. Four types of leisure foods—fried peanuts, fried pine nuts, roasted bhatanmu, and fried sunflower seeds—were tested using this method.

## 2 Materials and methods

### 2.1 Materials and reagents

Four types of leisure foods—fried peanuts, fried pine nuts, roasted bhatanmu, and stir-fried sunflower seeds cooked in corn oil—were purchased from a market in China.

The purity of sodium saccharin standard (concentration 1 mg/ml) was ≥99% and was procured from Shanghai Amperex Standard Technical Service Co., Ltd., Shanghai, China. *o*-sulfamoylbenzoic acid (analytical purity, purity >99.0%) was sourced from Shanghai Macklin Biochemical Technology Co., Shanghai, China. Potassium ferricyanide and zinc sulfate (analytically pure) were purchased from Sinopharm Chemical Reagent Co.

### 2.2 Instruments and chromatographic separation

Samples were detected using an HPLC-UV detector with an Agilent 1260 (Agilent Technologies Inc., USA). HPLC analysis was performed on a 5 μm 4.6 × 150 mm Eclipse XDB-C18 column (Agilent) with the mobile phase of acetonitrile (solvent A) and −0.1% formic acid aqueous solution (solvent B) at a flow rate of 1.2 ml/min, injection volume of 10 μl, and column temperature of 25 °C. Gradient elution was performed according to the program described in Table 1.

**Table 1:** Gradient elution schedule.

**Time/ min**	**(Solvent A) acetonitrile (%)**	**(Solvent B) 0.1% formic acid aqueous solution (%)**
0	8	92
10	52	48
10.1	8	92
15	8	92

### 2.3 Preparation of solutions

Sodium saccharin standard stock solution: 1 ml of sodium saccharin standard solution (1 mg/ml) was accurately drawn to 10 ml in a volumetric flask to prepare a standard stock solution of sodium saccharin at a concentration of 100 μg/ml.

*o*-sulfamoylbenzoic acid standard stock solution: 2 mg of *o*-sulfamoylbenzoic acid standard was accurately weighed, dissolved in primary water, and then diluted to 2 ml with a volumetric flask to prepare the standard stock solution of *o*-sulfamoylbenzoic acid at a concentration of 1 mg/ml. Then, 1 ml of the configured *o*-sulfamoylbenzoic acid solution (1 mg/ml) was carefully measured and diluted to a final volume of 10 ml using a volumetric flask. This results in an *o*-sulfamoylbenzoic acid solution with a concentration of 100 μg/ml that needs to be reconfigured in each experiment and stored at 4 °C under, protected from light.

Mixed standard solution preparation: 100 μl of each of the sodium saccharin standard reserve solution and *o*-sulfamoylbenzoic acid reserve solution (both at a concentration of 1 mg/ml) was accurately measured and made into a mixture of sodium saccharin and *o*-sulfamoylbenzoic acid standard solution (both at a concentration of 100 μg/ml) by adding a constant volume of primary water to 1 ml.

### 2.4 Experimental methodology

#### 2.4.1 Ultraviolet wavelength determination

The UV absorption wavelengths of sodium saccharin and *o*-sulfamoylbenzoic acid standard stock solutions were measured using a UV spectrophotometer. The two solutions to be measured were transferred to a cuvette for online detection, and the maximum absorption wavelength was determined to be 264 nm after full-wavelength scanning of sodium saccharin and *o*-sulfamoylbenzoic acid standard stock solutions using high-performance liquid chromatography DAD.

#### 2.4.2 Sodium saccharin frying experiment

For this experiment, 0.1 ml of 100 μg/ml of sodium saccharin standard reserve solution was pipetted and mixed thoroughly with 1 ml of corn cooking oil in a test tube. Then, the test tubes were placed in a constant-temperature sand bath instrument at different frying temperatures (160, 170, 180, 190, and 200 °C) while the frying time was maintained. Alternatively, changing frying times, the test tubes were placed in a constant temperature sand bath instrument, and frying times were adjusted to 10, 20, 30, 40, and 50 min, while maintaining a constant frying temperature. According to the national standard GB 5009.97 (China), after pretreating the sample from high-temperature frying, the processed products containing sodium saccharin were ready for machine testing.

#### 2.4.3 Sample preparation

Sample I: 1 ml of corn oil that cannot be used.

Sample II: this sample was prepared by placing sample I in a sand bath instrument to process at a frying temperature of 190 °C for 40 min.

Sample III: 0.1 ml of 100 μg/ml sodium saccharin standard reserve solution was pipetted and mixed with 1 ml of corn oil, which cannot be used for thorough mixing.

Sample IV: sample III was placed in a sand bath and processed at a frying temperature of 190 °C for 40 min.

Sample V: 0.1 ml of 100 μg/ml *o*-sulfamoylbenzoic acid standard stock solution was pipetted and mixed with 1 ml of corn oil, which cannot be used.

Sample VI: sample V was placed in a sand bath instrument and processed at a frying temperature of 190 °C for 40 min.

### 2.5 Standard curves

Standard solutions of sodium saccharin at concentrations of 0.0, 10, 20, 50, and 100 μg/ml were pipetted into separate containers. Then, the test solution was determined by following the HPLC conditions described in Section 2.2. The peak area for each standard solution was calculated. The standard curve was plotted using the mass concentration of sodium saccharin (μg/ml) as the horizontal coordinate and the peak area as the vertical coordinate, and the correlation coefficient was calculated. The standard curve of *o*-sulfamoylbenzoic acid was established similarly to that of sodium saccharin.

## 3 Results and discussion

### 3.1 Frying experiments

#### 3.1.1 Sodium saccharin frying test

**Figure 1:**
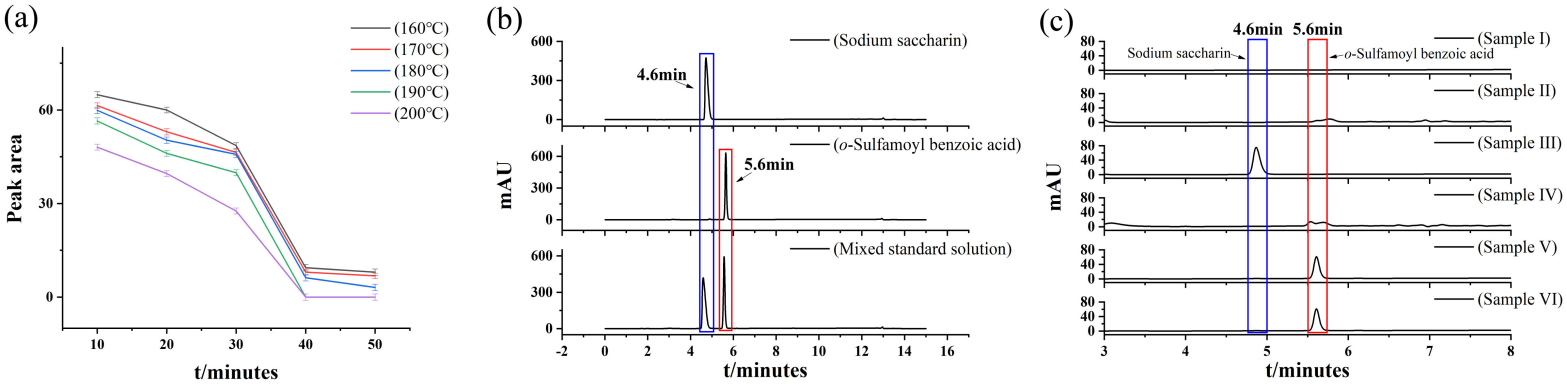
**(a)** Effect of frying time and temperature for sodium saccharin, **(b)** Chromatograms of sodium saccharin, *o*-sulfamoylbenzoic acid, and mixed standard solution, and **(c)** samples I, II, III, IV, V, and VI by frying.

The sodium saccharin standard was processed, according to the method in Section 2.4.2, in corn oil, and the results are shown in Figure 1a. Upon increasing the frying time within the high-temperature frying temperature range of 160–180 °C, changes in the content of the sodium saccharin standard were not significant. When the frying temperature exceeded 190 °C, the content of sodium saccharin began to decrease. The sodium saccharin standard was subjected to high-temperature frying experiments, and it was found that its content decreased with an increase in frying temperature; the content also decreased with an increase in frying time. Sodium saccharin can be found to be completely decomposed at 190 °C for 40 min by frying.

#### 3.1.2 Determination of target peaks

According to Figure 1b, standard solutions of sodium saccharin and *o*-sulfamoylbenzoic acid at different concentrations were analyzed and detected according to the method described in Section 2.2. The retention time of the two substances at different concentrations is obtained using a calibrated HPLC instrument to ensure that the determination results of sodium saccharin and *o*-sulfamoylbenzoic acid would not interfere with each other and to avoid the peak drift phenomenon. The mixed standard solution of sodium saccharin and *o*-sulfamoylbenzoic acid was also examined, and the peak times of both substances were found to be consistent with the peak times of the above expressions. Sodium saccharin exhibited a peak at 4.6 min and *o*-sulfamoylbenzoic acid exhibited a peak at 5.6 min.

#### 3.1.3 Sample controls

The prepared samples I, II, III, IV, V, and VI were tested by HPLC under the conditions described in Section 2.2. As shown in Figure 1c, Samples I and II did not interfere with the detection results of samples III, IV, V, and VI. There was a significant difference between the liquid chromatograms of samples III and IV, with sample III being treated by frying to reduce the content and produce new substances. The peak times of sample IV and sample V were identical at 5.6 min; the decomposition product of Sample III was determined to be *o*-sulfamoylbenzoic acid. The content of sample VI did not change after frying. It was determined that *o*-sulfamoylbenzoic acid was not affected by frying conditions. As shown in Figure 1c, when the sodium saccharin standard was treated under frying conditions (190 °C for 40 min), a decomposition product appeared. The peak time of the decomposition product was consistent with that of *o*-sulfamoylbenzoic acid, and the decomposition product was identified as *o*-sulfamoylbenzoic acid.

### 3.2 Determination of UV wavelength

**Figure 2:**
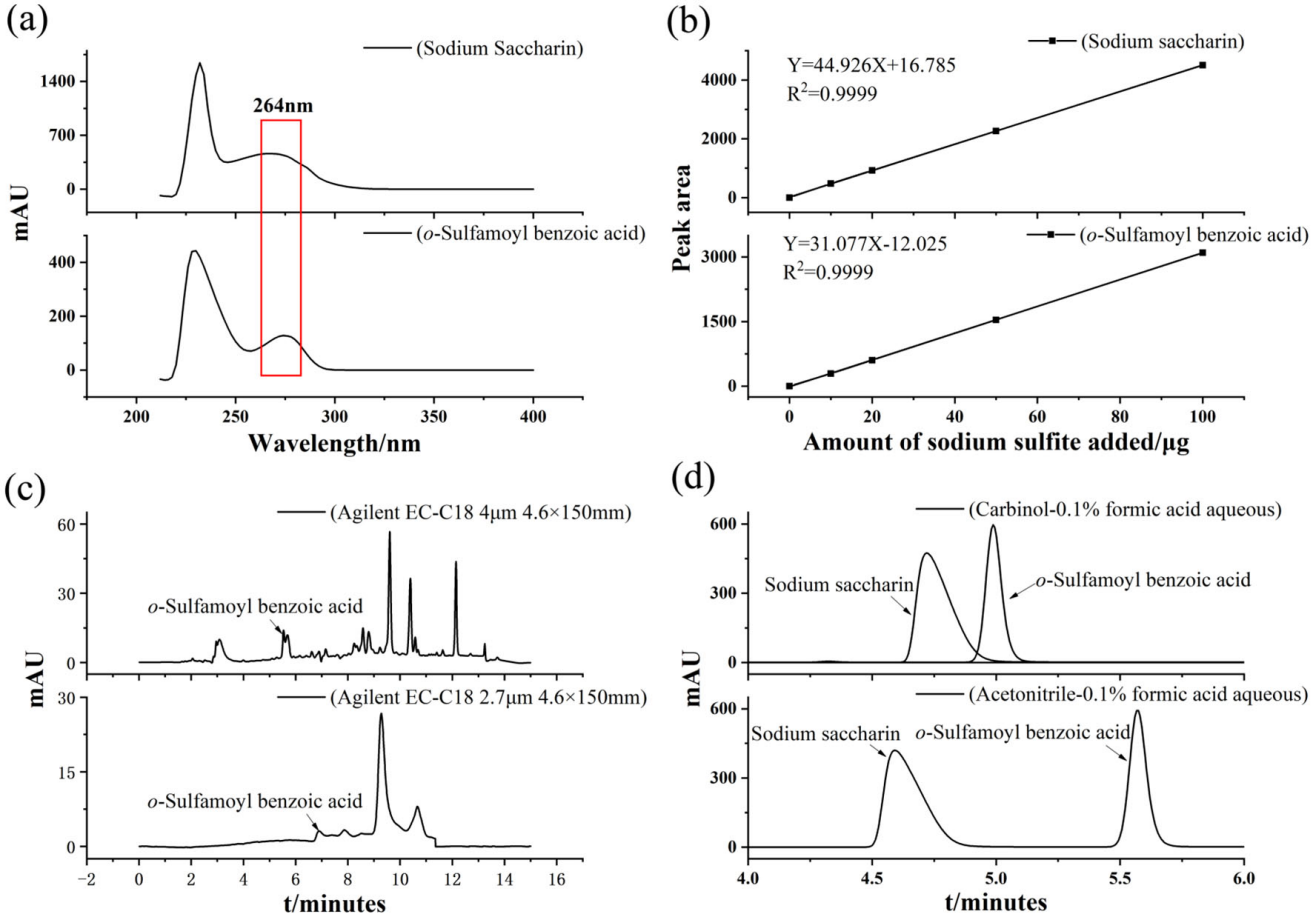
**(a)** Spectra of sodium saccharin and *o*-sulfamoylbenzoic acid **(b)** Standard curve for sodium saccharin and *o*-sulfamoylbenzoic acid **(c)** Chromatograms of sample IV detected by (Agilent 1260) HPLC columns Agilent EC-C18 4 μm 4.6 × 150 mm and Agilent EC-C18 2.7 μm 4.6 × 150 mm, and **(d)** Effect of the mobile phase system.

Full-band DAD scanning of sodium saccharin and *o*-sulfamoylbenzoic acid was performed according to the method described in Section 2.4.1. Figure 2a shows the chromatograms of four samples: sample I, sodium saccharin, *o*-sulfamoylbenzoic acid, and the mixed standard solution. As seen in the figure, compared with sample I, sodium saccharin peaked at a retention time of 4.6 min, *o*-sulfamoylbenzoic acid had a significant peak at 5.6 min, and the mixed standard had significant peaks at retention times of 4.6 and 5.6 min. The interference of Sample I with sodium saccharin and *o*-sulfamoylbenzoic acid was excluded. It was also determined that the peaks of sodium saccharin and *o*-sulfamoylbenzoic acid did not interfere with each other. It showed the spectra of sodium saccharin and *o*-sulfamoylbenzoic acid. The figure clearly shows that the maximum spectral peak areas appeared at 264 nm, and the best separation was achieved for both substances in Figure 2a.

### 3.3 Establishment of standard curves

#### 3.3.1 Establishment of sodium saccharin standard curve

The standard curve of the sodium saccharin standard was established according to the method in Section 2.5 (Figure 2b), using the peak area as the ordinate and the amount of standard sodium saccharin added as the abscissa. The linear equation for sodium saccharin is *Y* = 44.926*X* + 16.785, *R*^2^ = 0.9999. In the range of 0–100 μg/ml, the peak area of sodium saccharin showed a good linear relationship with the concentration of the corresponding standard solution.

#### 3.3.2 Establishment of a standard curve for *o*-sulfamoylbenzoic acid

The standard curve of the *o*-sulfamoylbenzoic acid standard was established according to the method in Section 2.5 (Figure 2b), using the peak area as the ordinate and the amount of standard *o*-sulfamoylbenzoic acid added as the abscissa. The linear equation for *o*-sulfamoylbenzoic acid is *Y* = 31.077*X* – 12.025, *R*^2^ = 0.9999. The peak areas of *o*-sulfamoylbenzoic acid, a decomposition product of sodium saccharin, showed good linearity with the concentrations of the corresponding standard solutions in the range of 0–100 μg/ml.

### 3.4 Chromatographic conditions

#### 3.4.1 Comparison of chromatographic columns

Sample III was placed in a sand bath instrument. Sample III was fried at 190 °C for 40 min. They were detected by Agilent 1260 HPLC columns equipped with Agilent EC-C18 (4 μm, 4.6 × 150 mm) and Agilent EC-C18 (2.7 μm, 4.6 × 150 mm). As shown in Figure 2d, the decomposition product of sample III (*o*-sulfamoylbenzoic acid) could not be effectively separated from the heterogeneous peaks. From Figure 2c, it was found that the decomposition product of sample III (*o*-sulfamoylbenzoic acid) could be effectively separated from the hetero peaks with a better response. Based on the above analysis, if the focus is on detection efficiency and cost, then an ordinary column was selected; if the focus is on high separation, then a high-performance chromatographic column was opted. In this study, an Agilent EC-C18 4 μm 4.6 × 150 mm column was used to establish the detection method for the samples.

#### 3.4.2 Mobile phase selection

Acetonitrile was selected as the organic phase because it separates compounds better than methanol and ethanol and has a lower baseline noise. In the aqueous phase, the effect of pure water and 0.1% formic acid aqueous solution systems on separation was investigated. As shown in Figure 2d, when a 0.1% formic acid aqueous solution and methanol were selected as the mobile phases, peak aggregation and poor separation occurred. As shown in Figure 2d, when a 0.1% formic acid aqueous solution and acetonitrile were used as the mobile phase system, acetonitrile had a stronger elution ability than methanol. This resulted in improved separation of the target substances. Therefore, the mobile phase selected for this process was an acetonitrile-0.1% formic acid aqueous solution.

#### 3.4.3 Elution method

Experimentally selected gradient elution separated the target peaks. The ratio of the mobile phase and elution rate was continuously adjusted according to the degree of elution. Then, the ratio of gradient elution was continuously adjusted according to the observation of the separation degree of the chromatogram, and the gradient elution conditions were finally determined. The liquid phase gradient elution conditions are shown in Table 1.

#### 3.4.4 Flow rate

When the mobile phase flow rate is gradually reduced, the separation degree increases, and the peak shape is stable and improved to a small extent; however, it also leads to the prolongation of the peak time of the target substance, which increases the detection and time costs. Taking the above considerations into account, a flow rate of 1.2 ml/min was adopted to effectively improve the detection efficiency and reduce the detection cost.

### 3.5 Determination of detection limits

Samples IV, V, and VI were tested according to the conditions outlined in Section 2.2, and the results are shown in Figure 1c. The unknown target peak appeared at a retention time of 5.6 min in the sample IV chromatogram, which was the same as the peak in the *o*-sulfamoylbenzoic acid chromatogram (sample V). It was indicated that the *o*-sulfamoylbenzoic acid substance did not decompose under the conditions of a frying temperature of 190 °C and frying for 40 min. The spiked experiments were carried out by taking 10 μg/ml and 100 μg/ml mixed standard solutions of sodium saccharin and *o*-sulfamoylbenzoic acid, respectively, and detected under the conditions described in Section 2.2. The analysis of HPLC detection of sample IV confirmed that the decomposition product of the sodium saccharin in the conditions of the high-temperature frying temperature of 190 °C for 40 min was *o-*sulfamoylbenzoic acid.

**Figure 3:**
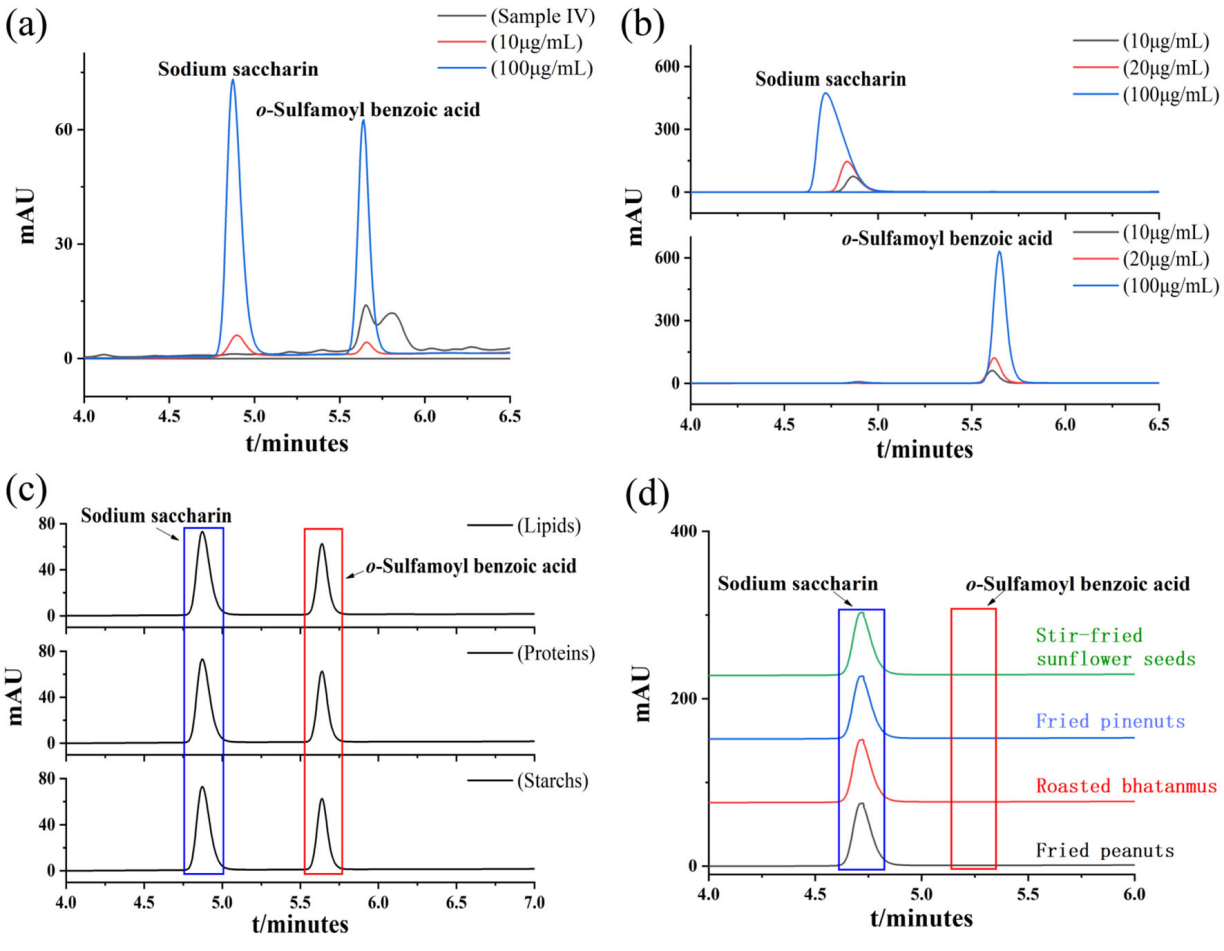
**(a)** Chromatograms of LOD and LOQ of sodium saccharin and *o*-sulfamoylbenzoic acid **(b)** Spiked recoveries and precision of sodium saccharin and *o*-sulfamoylbenzoic acid **(c)** Chromatograms of matrix stabilization experiments, and **(d)** Chromatograms of actual samples.

Samples III and V were processed, analyzed, and tested according to the research methods mentioned in Sections 2.2 and 2.4.2, and the results are shown in Figure 3a. The limit of detection (LOD) for sodium saccharin was calculated as 0.045 mg/kg based on a threefold signal-to-noise ratio (S/N = 3), and the limit of quantification (LOQ) was calculated as 0.135 mg/kg based on a 10-fold signal-to-noise ratio (S/N = 10). The limit of detection (LOD) for *o*-sulfamoylbenzoic acid was calculated to be 0.056 mg/kg based on a threefold signal-to-noise ratio (S/N = 3), and the limit of quantification (LOQ) was calculated to be 0.167 mg/kg based on a 10-fold signal-to-noise ratio (S/N = 10). This indicates that the method has good sensitivity.

### 3.6 Recovery experiments and precision

Three mass concentration levels of the sodium saccharin standard solution were added according to the method described in Section 2.2, with the addition range of 10–100 μg/ml, and the spiked amounts were 10, 20, and 100 μg/ml, respectively, and the results are shown in Table 2 and Figure 3b. *o*-sulfamoylbenzoic acid was subjected to the same spiked recovery test as that for sodium saccharin, and the results are shown in Table 2 and Figure 3d. Each sample was prepared six times in parallel for determination, and the spiked recoveries were calculated using the formula (Equation 1). In Equation 1, *P* denotes the spiked recovery, and *C*_1_ represents the total mass of sodium saccharin when the standard solution of sodium saccharin was added to the product of sodium saccharin after frying. *C*_2_ refers to the mass of sodium saccharin. The spiked recoveries of *o*-sulfamoylbenzoic acid were calculated as Equation 1. The relative standard deviations of sodium saccharin after frying and *o*-sulfamoylbenzoic acid were calculated using the formula (Equation 2). Sodium saccharin spiked with a recovery rate in the range of 105.67%112.16% with relative standard deviations (RSDs) in the range of 0.24%−1.39%. The recovery rate of *o*-sulfamoylbenzoic acid spiked to 98.67%−108.16% with relative standard deviations (RSDs) in the range of 0.13%−1.51%. The results obtained by using the experimental analytical method showed that the method had good precision and high accuracy.


(1) $$P=\frac{c_{1}-c_{2}}{c_{3}}\times 100\%$$

(2) $$RSD=\frac{SD}{X}\times 100\%$$

**Table 2:** Spiked recovery rate and precision.

**Samples**	**Add levels/(mg/kg)**		**Detection value/ (mg/kg)**						**Blank/ mg/kg**	**Recovery rate/%**	**RSD/ %**
			**1**	**2**	**3**	**4**	**5**	**6**			
Sample I	10	Sodium saccharin	10.567	10.499	10.471	10.633	10.569	10.664	0	105.67	0.24
	20		22.531	20.668	21.021	21.886	22.067	21.811	0	108.32	1.39
	100		112.685	111.96	113.94	111.991	110.667	111.717	0	112.16	0.65
Sodium saccharin	10	*o*-sulfamoylbenzoic acid	9.862	10.265	9.768	9.942	9.766	9.838	0	98.67	0.13
	20		19.835	20.004	19.894	20.461	20.884	20.506	0	101.32	1.51
	100		107.364	108.531	107.253	109.541	109.838	106.433	0	108.16	0.69

### 3.7 Matrix stabilization experiments

To examine the stability of two substances, sodium saccharin and *o*-sulfamoylbenzoic acid, stability tests with different matrices were carried out. Considering the complexity of the substances in the measured food samples, the food samples and standard solutions were examined separately to avoid influencing the test results. The pre-treatment followed the national standard GB 5009.28-2016, and the treated sample solution was stored at 4 °C under the condition of light protection. The samples were injected and measured at intervals of 1 h according to the conditions described in Section 2.2 to explore the stability of the situation. The data obtained were analyzed, and the results for the compounds that remained stable within 10 h of the test are shown in Table 3. The two substances, sodium saccharin and *o*-sulfamoylbenzoic acid, were stable in fried peanuts, roasted bhatanmu, fried pine nuts, and stir-fried sunflower seeds.

**Table 3:** Matrix stabilization experiments 1–10 h (as indexed by peak area).

**Time/h**	**1**	**2**	**3**	**4**	**5**	**6**	**7**	**8**	**9**	**10**
Sodium saccharin	98.32	99.83	100.04	99.33	98.96	100.03	99.17	100.33	99.34	99.68
*o*-sulfamoylbenzoic acid	101.1	101.23	100.79	100.93	101.61	100.86	101.02	100.86	101.1	101.15
Fried peanuts	10.67	9.89	11.13	11.04	10.92	10.81	9.97	10.96	10.63	11.11
Roasted bhatanmu	10.53	10.63	11.08	11.13	10.92	10.98	10.51	11.01	10.31	11.21
Fried pine nuts	13.34	12.87	13.13	12.78	13.09	12.11	13.16	13.22	12.63	12.66
Stir-fried sunflower seeds	11.71	11.93	12.03	10.11	12.62	11.08	10.91	11.46	11.24	10.04

### 3.8 Matrix effect evaluation method

The matrix effect was calculated from the results of HPLC detection, respectively, using the peak area as an indicator, and the specific formula for calculating the matrix effect (ME) based on the peak area ratio is shown in Equation 3, which indicates that the matrix has an enhanced effect when ME > 100%. When ME <100%, the matrix exhibited a weakening effect. When ME = 100%, the matrix interference is negligible. It is generally believed that 80% <ME <120% indicates that the matrix effect is not significant, while when ME > 120% or ME <80%, the analysis showed a stronger matrix effect, two and two control experimental matrices, a total of three groups (starch/lipid, starch/protein, and lipid/protein), and the combined results of the calculations are shown in Table 4 and Figure 3c. The available data were all within the range of 80% <ME <120%. It can also be observed from the data that the different matrices did not have a significant effect on the available data.


(3) ME=(Am/As)×100%

**Table 4:** Matrix effect calculation results.

**Samples**	**Peak area (ME%) /HPLC**
Starches/lipids	101.32/99.66
Starches/proteins	103.41/100.79
Lipids/proteins	97.81/99.55

### 3.9 Actual sample test

In this study, the above-established HPLC method was used to analyze the contents of sodium saccharin and *o*-sulfamoylbenzoic acid in commercially available recreational food products, such as fried peanuts, roasted bhatanmu, fried pine nuts, and stir-fried sunflower seeds. Two parallel samples were used for the analysis. The results are shown in Table 5 and Figure 3d. Sodium saccharin was detected, while *o*-sulfamoylbenzoic acid was not detected.

**Table 5:** Results of the actual samples.

**Sample**	**Detection value/(g/kg)**				**Sample**	**Detection value/(g/kg)**			
Stir-fried sunflower seeds−1	Sodium saccharin	0.770	*o*-sulfamoylbenzoic acid	Undetected	Stir-fried sunflower seeds−2	Sodium saccharin	0.761	*o*-sulfamoylbenzoic acid	Undetected
Fried pinenuts−1		0.510		Undetected	Fried pinenuts−2		0.536		Undetected
Roasted barnacles−1		0.224		Undetected	Roasted barnacles−2		0.230		Undetected
Fried peanuts−1		0.620		Undetected	Fried peanuts−2		0.647		Undetected

## 4 Conclusion

In this study, the detection methods of sodium saccharin standard and *o*-sulfamoylbenzoic acid standard were established by HPLC. By HPLC, the peak time of sodium saccharin was 4.6 min, the detection limit was 0.045 mg/kg, the quantification limit was 0.135 mg/kg, the recovery rate was in the range of 105.67%−112.16%, and the relative standard deviations (RSDs) were in the range of 0.24–1.39%. The peak time of *o*-sulfamoylbenzoic acid was 5.6 min, the limit of detection (LOD) was 0.056 mg/kg, the limit of quantification (LOQ) was 0.167 mg/kg, the recovery rate was in the range of 98.67%−108.16%, and the relative standard deviations (RSDs) were in the range of 0.13%−1.51%.

Sodium saccharin would have decomposition products of *o*-sulfamoylbenzoic acid after a frying temperature of 190 °C with a frying time of 40 min. Sodium saccharin and its decomposition product, *o*-sulfamoylbenzoic acid, were detected and determined by the established HPLC method. The established detection method was applied to analyze commercially available fried leisure foods, such as fried peanuts, fried pine nuts, roasted bhatanmu, and stir-fried sunflower seeds (sodium saccharin was added to all four food labels), and all of them detected sodium saccharin, but not *o-*sulfamoylbenzoic acid.

Consumers are concerned about the nutritional value of food and food safety hazards. Sodium saccharin is an artificial sweetener added to food, and during repeated high-temperature frying over a long period, it thermally decomposes into *o*-sulfamoylbenzoic acid. In this study, only the detection method for sodium saccharin and *o*-sulfamoylbenzoic acid was established, and it was found and determined that *o*-sulfamoylbenzoic acid was indeed present in sodium saccharin under repeated high-temperature frying conditions. From the perspective of food safety considerations, sodium saccharin after repeated high-temperature frying should be considered, and the direct consumption of *o*-sulfamoylbenzoic acid, whether there are hazards, needs to be studied by relevant professionals.

## Data Availability

The original contributions presented in the study are included in the article/supplementary material, further inquiries can be directed to the corresponding authors.
